# Aroma Components in Horticultural Crops: Chemical Diversity and Usage of Metabolic Engineering for Industrial Applications

**DOI:** 10.3390/plants12091748

**Published:** 2023-04-24

**Authors:** Farhat Abbas, Yiwei Zhou, Dylan O’Neill Rothenberg, Intikhab Alam, Yanguo Ke, Hui-Cong Wang

**Affiliations:** 1 Key Laboratory of Biology and Genetic Improvement of Horticultural Crops-South China/Guangdong Litchi Engineering Research Center, College of Horticulture, South China Agricultural University, Guangzhou 510642, China; 2 Guangdong Key Laboratory of Ornamental Plant Germplasm Innovation and Utilization, Environmental Horticulture Research Institute, Guangdong Academy of Agricultural Sciences, Guangzhou 510642, China; 3 College of Economics and Management, College of Agriculture and Life Sciences, Yunnan Urban Agricultural Engineering & Technological Research Center, Kunming University, Kunming 650214, China

**Keywords:** volatile organic compounds, horticultural commodities, industrial applications, metabolic engineering

## Abstract

Plants produce an incredible variety of volatile organic compounds (VOCs) that assist the interactions with their environment, such as attracting pollinating insects and seed dispersers and defense against herbivores, pathogens, and parasites. Furthermore, VOCs have a significant economic impact on crop quality, as well as the beverage, food, perfume, cosmetics and pharmaceuticals industries. These VOCs are mainly classified as terpenoids, benzenoids/phenylpropanes, and fatty acid derivates. Fruits and vegetables are rich in minerals, vitamins, antioxidants, and dietary fiber, while aroma compounds play a major role in flavor and quality management of these horticultural commodities. Subtle shifts in aroma compounds can dramatically alter the flavor and texture of fruits and vegetables, altering their consumer appeal. Rapid innovations in -omics techniques have led to the isolation of genes encoding enzymes involved in the biosynthesis of several volatiles, which has aided to our comprehension of the regulatory molecular pathways involved in VOC production. The present review focuses on the significance of aroma volatiles to the flavor and aroma profile of horticultural crops and addresses the industrial applications of plant-derived volatile terpenoids, particularly in food and beverages, pharmaceuticals, cosmetics, and biofuel industries. Additionally, the methodological constraints and complexities that limit the transition from gene selection to host organisms and from laboratories to practical implementation are discussed, along with metabolic engineering’s potential for enhancing terpenoids volatile production at the industrial level.

## 1. Introduction

Floral scent and color are essential traits for many floricultural crops, and floral volatiles are biologically and economically significant plant-derived substances that play a vital role in pollinator attraction, defense mechanisms, and interaction with the surrounding environment [1,2,3]. Floral volatiles have a low molecular weight and are lipophilic, derived from biosynthetic pathways, including terpenoid, phenylpropanoid/benzenoid, and fatty acids [4,5]. Volatile terpenoids are among the most abundant volatile organic compounds (VOCs), followed by selective benzenoids/phenylpropanoids.

Fruits and vegetables generate a variety of volatile compounds that contribute to their distinctive aromas and flavor. Aroma has received increased attention in recent years as an important characteristic of fruit quality. The most common volatile compounds found in fruits are esters, aldehydes, alcohols, lactones, ketones, terpenoids, and apocarotenoids, which determine the differences in aromas [6]. Fruit quality encompasses both preharvest developments, such as changes in flavor, color, and texture with fruits development, and postharvest maintenance as perishable tissues age [7]. Flavor is composed of both the perception in the mouth (sweetness, acidity, or bitterness) and the aroma, which is synthesized by numerous volatile compounds [8]. The insight into the key volatile flavor enzymes that hold the distinctiveness of the natural fruit is critical, because it produces the fruit’s primary sensory uniqueness and peculiar flavor.

Terpenoids are derived from five-carbon isoprene units, including monoterpenes, sesquiterpenes, apocarotenoids, and many others [1,9]. Five-carbon unit (C5) metabolites such as isopentenyl diphosphate (IPP) and dimethylallyl diphosphate (DMAPP) are precursors for terpene biosynthesis and are produced in distinct cellular locations by the methylerythritol phosphate (MEP) and mevalonic acid (MVA) pathways, respectively [9]. Likewise, more than 8000 phenylpropanoids metabolites have been identified [10]; benzenoids are the second largest class of VOCs generated from the amino acid phenylalanine, whereas fatty and amino acid are crucial VOCs found in fruit and flower aromas [8]. Benzenoids/phenylpropanoids are generated via shikimate pathways via the catalyzation of phenylalanine [4]. Meanwhile, the lipoxygenase (LOX) pathway is responsible for the catabolism of fatty acids, the primary precursors of volatile components in floral scents and fruit aromas [8]. Several key terpenoids biosynthesis genes and transcription factors have been identified from various plant species [11,12,13,14,15,16,17].

VOCs have a huge impact on human society, due to their applications in the food, cosmetics, and pharmaceutical industries. They are used in the pharmaceutical and food industries due to their effectiveness and potential as medicinal preservatives and flavor enhancers [4,18,19]. Terpenes are found in a wide range of products, such as rubber, pyrethrin-based insecticides, carvone and hecogenin-based detergents, caryophyllene-based antihistamines and antibiotics, methanol-based cleaning agents, and many others [17,20,21]. Similarly, several important crops, such as blueberries, apples, litchi, and cucurbits, rely heavily on volatiles for pollination [22,23,24].

Rapid advancements have been made in floral aroma engineering for terpenoid biosynthesis in model plants due to their commercial and ecological significance. The discovery of key structural genes, as well as the decoding of the biosynthesis pathway and enzyme proteins associated with these pathways, has made genetic engineering in plants exceptionally feasible [4,16,25]. Several input and output attributes in crops can be improved via genetic engineering approaches, such as weed management through allelopathy, insect/pathogen resistance, intensifying the aroma production of fruits and vegetables (by modifying the floral fragrance), and the output of bioactive compounds. The industrial use of volatile terpenoids is widespread, and they have promising economic prospects; however, there are several obstacles that limit large-scale production. These objectives are now extremely attainable due to the rapid advances in multi-omics technologies.

Multiple studies on floral scents and fruit aromas have advanced our understanding of their roles, components, biosynthesis, and regulation over the last decade. Furthermore, prior studies focused on floral scents or fruit aromas individually, even though both have significant volatile components that contribute to the economic value of horticultural crops. More emphasis was placed on aroma compounds and their role in the environment rather than human health and industrial applications. In this review, we emphasized the role of volatile organic compounds in the aroma quality of fruits and vegetables, as well as their industrial applications. Furthermore, the complexity and limitations of aroma production on an industrial scale, as well as the application of various metabolic engineering approaches, are discussed. Herein, we place more emphasis on volatile terpenoids, because they are the most profuse class of volatile organic compounds.

## 2. Aroma Profile of Horticultural Crops 

Volatiles released from flowers, fruit, and vegetable crops act as pollinator attractants, repel herbivores, and are essential for plant defense [26]. Plants emit multiple types of volatiles from their flowers, roots, stems, leaves, seeds, barks, fruits, and other special storage parts as part of their metabolic activity during the developmental stages [12,27]. Even though many aroma characteristics of various crops are shared, each crop has a peculiar aroma that is determined by the mixture of volatiles, the concentration, and the perspective threshold of individual compounds.

Fruit and vegetable quality parameters consist of both pre- and postharvest development. Preharvest development includes changes in color, flavor, and texture with fruit development, growth, and ripening, while postharvest maintenance affects how perishable tissues age [28,29]. Flavor is based on the perception in the mouth (sweetness, acidity, or bitterness), as well as the odor, which is generated by many volatile compounds. Since aroma is among the most valuable features, volatile flavor compounds play crucial roles in determining perceptions of customers and the acceptability of products [30]. As a result, VOCs are regarded as biomarkers in horticultural commodity quality management, and insight into the key volatile flavor metabolites that carry the distinctive characters of the natural fruit is critical [31]. Many factors influence the composition of these VOCs, such as genetic make-up, degree of maturity, environmental conditions, and postharvest handling and storage. Herein, we summarize couple of important horticultural crops and their aroma volatile profiles.

### 2.1. Aroma Profile of Fruits

Fruit aroma is an important factor in determining fruit quality. VOCs that contribute to the distinctive aromas and flavors of fruits and vegetables most commonly include, esters, terpenoids, alcohols, lactones, aldehydes, ketones, and apocarotenoids. Volatile profiles are complex, because they depend on the cultivar; ripeness; pre- and post-harvest climatic parameters; fruit samples (whole fruit, slices, or homogenized samples); and analytical methods employed (Tiwari et al. 2020). For example, the number, identity, and intensity of VOCs released by ripening apple fruit vary according to cultivar. Moreover, environmental and agronomic conditions, the stage of maturity, handling and storage, and the amount of time exposed to UV radiation have all been shown in various studies to have significant influence on the amount of these compounds [32]. 

**Figure 1:**
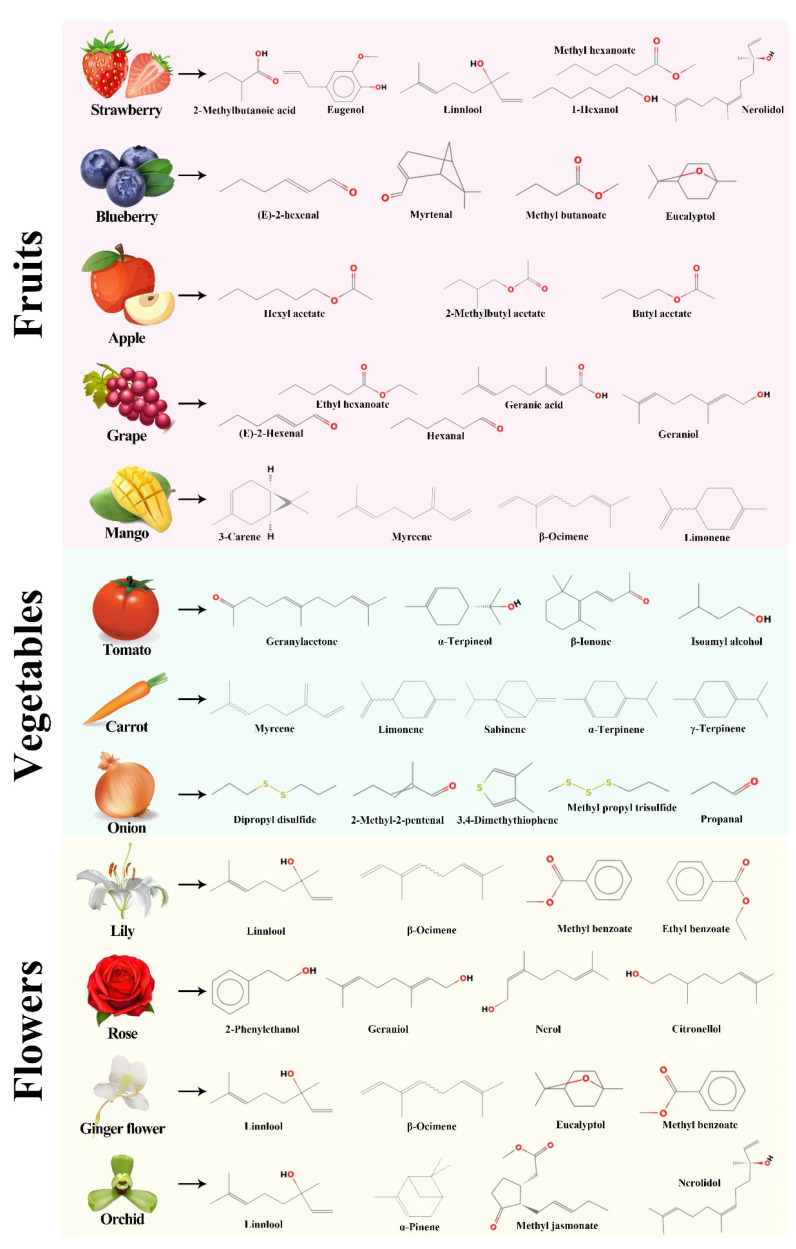
A volatile diversity of important fruits, vegetables, and flowers.

The aromatic compounds are frequently emitted when enzymes and precursors/substrates are intact. Although a large variety of chemical compounds have been identified as volatile compounds in fresh fruit, yet a subset of these compounds has been found as impact elements of fruit flavor, depending on their abundance and olfactory thresholds [6]. Monoterpenes and sesquiterpenes are among the most predominant group of compounds in the aroma profile. These compounds can also play a significant role in determining the odor profile. The significant influence of aroma on fruit marketability motivates the need to advance in our comprehension of this quality trait (Figure 1). Berry fruits and pomaceous fruits are two prominent fruit families with excellent nutritional contents. Berry fruits, such as strawberries, blueberries, raspberries, and grapes, are commercially well known for their sweet flavor, which is attributed to fructose and volatile compounds. Apples, citrus, peaches, and mangos are examples of pomaceous fruits which volatile chemicals have been thoroughly investigated in numerous cultivars.

Strawberries (*Fragaria* x *ananassa*) are by far the most popular berry fruit crop worldwide, valued for its distinct flavor and nutritional content, and its overall likeability is highly influenced by the sensory characteristics, sweetness, and flavor intensity [33,34]. Strawberry aroma is an excellent example of a complex fruit aroma, and VOCs are essential components of strawberry flavor, despite accounting for less than 0.01% of the fruit’s weight [34]. Fresh strawberries have been found to contain over 360 volatile compounds, including esters, alcohols, ketones, furans, terpenes, aldehydes, and sulfur compounds; however, their concentration and composition vary depending on cultivar and maturity [35,36]. Nine sesquiterpenoids and three triterpenoids were revealed to have been isolated from ‘Falandi’ strawberries as nonphenolic components with antidiabetic, antitumor, and antioxidant properties [37,38]. Even though volatile compounds account for only 0.001%–0.01% of the fruit’s weight, they are critical elements of strawberry flavor, and minor changes can drastically alter the taste [34].

Apples (*Malus domestica*) are one of the most popular fruits, and their distinct aroma is caused by a complex mixture of volatiles that varies depending on the volatile compounds, concentrations, and odor thresholds. Apples are low-calorie fruits that are exceptionally high in vitamins, minerals, acids, dietary fiber, and phenols [39]. Moreover, apples are beneficial in the prevention of several cardiovascular diseases, cancer, and asthma [40]. In addition, the phenolic compounds and triterpene acids found in apples have anti-inflammatory properties and have been shown to provide protection against Alzheimer’s disease [41]. In apples, 300 VOCs were found, and each compound contributed uniquely to the scent profile [42,43]. Among terpenoids, α-farnesene, D-limonene, geranyl acetone, and farnesol are prevalent, but their content varies with variety [41,44]. In addition, monoterpenes, sesquiterpenes, and various terpene derivatives have been identified in apple floral and vegetative tissues.

Blueberries (*Vaccinium* spp.) are the second most valuable soft fruit species after strawberries, and their aroma is determined by the interaction of large numbers of VOCs produced by the fruit during ripening [45,46]. Even though ripening has a major impact on blueberry aroma, the wide genetic distinctions among blueberry species also contribute to the wide range of blueberry aroma profiles. For example, a large production of esters (i.e., methyl acetate, ethyl acetate, or methyl butanoate) is characteristic of lowbush blueberry (*V*. *angustifolium*), bilberry (*V*. *myrtillus*), and other wild species, whereas highbush (*V*. *corymbosum*) and rabbiteye blueberry (*V*. *virgatum*) profiles are typically associated with a high composition of (*E*)-2-hexenal, hexanal, and (*Z*)-3-hexenol and terpene alcohols such as linalool, nerol, and geraniol [47,48,49]. Eight terpenoid volatiles have been identified as the primary metabolic group linked to these olfactory perceptions. These include *p*-cymene, myrtenal, linalool, L-carvenol, geranyl acetone, geranyl acetate, D-limonene, and myrcene [49,50].

Similarly, more than 200 volatile compounds have been identified in raspberries (*Rubus idaeus* x *ursinus*), with terpenoids contributing significantly to the raspberry aroma profile [51]. The primary volatile chemicals in the volatile profile of raspberry include raspberry ketone, linalool, α/*β*-ionone, nerol, (*E*, *Z*)-3-hexenol, geraniol, *β*-ocimene, *β*-pinene, *α*-terpineol, hexanal, 1-octanol, Furaneol, heptanal, benzaldehyde, and *β*-damascenone; however, among these, *α*/*β*-ionone, linalool, nerol, geraniol, and raspberry ketone may play a significant role in red raspberry aroma [6,51]. Further research revealed that the volatile composition varies from cultivar to cultivar, particularly *α/β*-ionone, linalool, geraniol, and (Z)-3-hexenol [52,53]. Likewise, the most abundant volatiles in the volatile profile of blackberries (*Rubus laciniata*) are *p*-cymen-8-ol, α-terpineol, 2-Heptanol, 4-terpineol, 2-heptanone, nonanal, pulegone, isoborneol, 1-octanol, elemicine, 1-hexanol, myrtenol, and carvone [54]. 

Grapes (*Vitis* spp.) are one of the extensively grown fruit crops in the world, and their distinct appearance and flavor have earned them a high level of commercial importance on a global scale [55]. Grapes are classified as wine grapes or table grapes based on their physiochemical properties and utilization. For wine grapes, taste refers to the ultimate product of grape fermentation and other industrial operations such as juicing [56,57]. Flavor is important in affecting customer acceptability of ripe fresh grapes, which is normally experienced as a combination of taste noticed in the mouth and aroma traveling into the nose. The fruit volatiles of *Vitis vinifera* contain a diverse range of compounds, including monoterpenes, C_13_ norisoprenoids, alcohols, esters, and carbonyls [58]. Each grape variety’s unique aroma is the result of a complex interaction between many different volatile compounds; for instance, more than 100 terpenoids have been identified as contributing to the aroma of Muscat-type grape varieties, and these are thought to be responsible for the distinctive varietal flavors of these table grapes [55,56]. 

Among the most widely grown and consumed fruits, *Citrus* is a vital part of the diets of people all over the world. In the citrus fruits, mandarins are among the most prominent representatives, because they, along with the pomelo and the citron, are thought to be the original predecessors from which all other citrus species evolved [59,60]. In *Citrus*, terpenoids such as *β*-pinene, *S*-linalool, valencene, and limonene are the primary aroma compounds. Terpenoids (d-limonene, valencene, linalool, terpinen-4-ol, and α-terpineol) were among the most rich and major constituents of aroma compounds and significantly contribute to the distinctive flavor of ‘Dortyol yerli’ orange juice, which contained 58 volatile components that were identified and quantified [61]. The juice of four citrus species—a ‘Powell’ Navel orange (*Citrus sinensis*), a ‘Clemenules’ mandarine (*C*. *reticulate*), a ‘Fortune’ mandarine (*C*. *reticulate*), and a ‘Chandler’ pummelo (*C*. *maxima*)—contains over a hundred volatile chemicals, with a few compounds unique to individual citrus varieties [6,61].

Peach (*Prunus persica*) volatiles have been extensively studied, and more than 100 volatile compounds responsible for the quality of peach aromas have been identified [62,63]. Alcohols, C_6_ compounds, esters, terpenoids, aldehydes, lactones, and ketones are the main volatile components of the peach fruit aroma. The most abundant peach volatiles are C_6_ compounds, linalool, esters, C_13_ norisoprenoids, benzaldehyde, and lactones. Peach fruit flavor is thought to be heavily influenced by esters such as hexyl acetate and (*Z*)-3-hexenyl acetate [6,64]. A previous finding demonstrated that the intensity and aroma composition of peach fruit differ depending on cultivar, fruit development, processing, and storage conditions [63,65,66]. Additionally, numerous studies have demonstrated the impact of culture conditions and management on the concentration and composition of aroma volatiles in peach fruit [6,65,67].

Mango (*Mangifera indica*) has a very appealing flavor, and more than 270 aroma volatile compounds in various mango varieties have been outlined in free form. Terpenes are the most abundant class of compounds in New World mangos, accounting for 16–90% of the total, while there are great variations in both quantity and quality of ketones, esters and alcohols, especially of Old-World varieties. Monoterpene 3-Carene is the most abundant compound in several New World mango varieties, followed by myrcene, β-ocimene, and limonene, whereas sesquiterpene hydrocarbons can be found in up to 10% of cultivars [68].

Recent discovery of health advantages in pomegranate (*Punica granatum*), together with its sensory quality and flavor preferences, have made it a commercially significant fruit [69,70]. Several different types of volatiles, such as alcohols, aldehydes, ketones, and terpenes, combine to create the aroma, which is characterized by a wide range of ‘green’, ‘woody’, ‘earthy’, ‘fruity’, ‘floral’, ‘sweet’, and ‘musty’ notes [71,72]. Volatiles discovered in pomegranate characteristics that contribute to the distinct scent and flavor include three alcohols, six aldehydes, one ketone, and eleven terpenes (six monoterpenes, two oxygenated terpenes and three sesquiterpenes). The majority of these compounds appeared to be hexanal derivatives (hexanal, hexanol, and (*Z*)3-hexenol) and terpenes (α-pinene, limonene, α-terpineol, and β-caryophyllene), all of which can be regarded as significant aroma volatiles in pomegranate fruits [71,73].

The volatile composition of fresh fruit is continually shifting owing to the complexity of the volatile profiles. Several factors determine volatile composition, such as fruit genetics, maturity, climate changes during fruit development, postharvest management, and storage. So far, our knowledge of the interaction between these factors that establishes the fruits volatile composition and flavor is confined and we need to explore further in order to improve the quality characteristics of fruits. Regulating volatile compound emissions is often hampered by our current lack of knowledge regarding the regulation of pathways leading to the synthesis of such compounds. Huge progress in metabolic engineering targeted at enhancing the set of volatiles emitted by fruits have been assisted by the characterization of key signature enzymes associated with biosynthesis pathways of flavor and aroma compounds in certain fruits. Marker-assisted selection based on QTL and linkage analyses can help to transfer desirable flavor and aroma from aromatic lines to non-scented or less aromatic lines, as well as promising agronomic traits.

### 2.2. Aroma Profile of Vegetables

Volatiles not only improve the quality of fruits and vegetables, but also have significant impacts on human health due to the numerous medicinal properties they possess. Recently, there has been an increase in global interest in plant-based foods, specifically fruits and vegetables, because of the ability of bioactive compounds to scavenge free radicals (such as, reactive oxygen and reactive nitrogen species), in addition to their antimicrobial, anti-inflammatory, and antiproliferative activities [74,75]. The occurrence of secondary metabolites, such as polyphenols, carotenoids, and terpenoids, in specific food matrices contribute to their functional properties, which are manifested as an enhanced counteractive action of the development of several chronic diseases, such as cardiovascular disease, cancer, neurodegenerative diseases, and diabetes [18,76]. Carrots, tomatoes, onions, and spinach are the most commonly utilized aromatic vegetables in the daily diets of various cuisine cultures around the world, and their volatile chemicals have been thoroughly explored.

Terpene compounds dominate the volatile profile of carrot (*Daucus carota*) varieties. Terpenes are abundant in orange carrot, with 31 identified that accounted for 58.1% of the total volatiles of this variety, whereas terpenes are less prevalent in white carrot, with 22 volatile metabolites identified that accounted for 61.3% of the total volatile compounds [31]. 

In terms of the volatile composition of tamarillo (*Solanum betaceum*), 65 volatile biomarkers were identified, 20 of those were terpenoid compounds, 17 esters, 7 alcohols, 5 benzyl compounds, 4 aldehydes, 4 furan compounds, and the rest 7 miscellaneous compounds. In tamarillo, 65 volatile metabolites were identified, including 20 terpenes, 17 esters, 7 alcohols, 5 benzenes, 4 aldehydes, 4 furans, and 7 various compounds [31].

The volatile profile of both (red and yellow) onion (*Allium cepa*) varieties is very distinct. Red and yellow onions have very different volatile profiles. Red onions contain aldehydes (26.7%), organosulfur compounds (19.6%), and carboxylic acids (15.3%), while yellow onions contain mainly organosulfur compounds (73.8%) [6,77]. 

A total of 61 volatile compounds were identified in *Beta vulgaris* (beet), the majority of which were terpenoid (61.0%), furanic (20.6%), carboxylic acids (5.6%), and benzene derivatives (5.2%) [31]. Similar to onions, garlic and broccoli are also rich in organosulfur compounds, which give it its aroma, flavors, and bioactive attributes [78,79].

In tomatoes (*Solanum lycopersicum*), aldehydes and furanic compounds make up 71% of the total volatile profile. The remainder aroma and taste contributors were alcohols (6.6%), esters (5.9%), terpenoid compounds (5.5%), carboxylic acids (4.7%), organosulfur compounds (2.9%), and ketones (1.6%) [31,80].

In spinach’s (*Spinacia oleracea*) volatile profile, 57 metabolites were identified, including 14 esters, 13 terpenes, 8 alcohols, and 7 aldehydes [31]. Other interesting functions in plants that are not discussed here include pathogen defense, heat and oxidative stress tolerance, signaling among plant organs, inter-plant interaction, and allelopathy [30,81,82].

Predicting and better understanding the behavior of flower visitors, as well as the function of volatile compounds in certain plants, requires a thorough quantitative and qualitative analysis of aroma compounds. The combination of solid-phase microextraction in headspace mode (HS-SPME) and gas chromatography-mass spectrometry (GC-MS) is a popular and effective analytical method for metabolomics studies of volatile organic compounds (VOCs) due to its many advantageous features; however, there is a need to explore further. Understanding the volatile profile of fruits and vegetables is useful for enhancing their flavor and determining which of their metabolites have the greatest biological significance and, thus, the most potential for application in targeted nutraceutical therapies.

### 2.3. Function and Volatile Profiles of Flowers

Flower fragrances are a complex mixture of volatiles that play multiple roles and are used by pollinators in combination with other signals such as color. Floral scent is broadly used in aromatherapy, perfume, cosmetics, flavoring, and pharmaceutical industries; however, their prime role is to facilitate pollinator, herbivore, and pathogen interactions in their native ecosystems [1,83]. Floral visitors utilize floral fragrance to predict the quantity of incentive found in flowers, to aid in the particular aspect host flower, or as chemically similar signals to those essential for pollinating insects in other ecological circumstances [26,84]. β-ocimene is universal terpenoid implicated in pollinators attraction such as trans-β-ocimene emitted in highest amount at night coinciding with flower opening and pollinators’ activity [85,86]. Floral scents are composed of a variety of compounds that are classified as terpenoids, phenylpropanoids/benzenoids, fatty acid derivatives. Terpenoids are the most diverse class of volatile compounds, with over 40,000 structures synthesized from C_5_ isoprene units, including monoterpenes, sesquiterpenes, apocarotenoids, and others [8]. Ginger flowers, jasmine, and Narcissus are aromatic plants with distinct fragrance properties, and their volatile chemicals have received a lot of interest in commercial applications in recent years.

Orchid is the richest flowering plant family (*Orchidaceae*), with 20,000–30,000 species, 75% of which are fragrant [87,88]. Monoterpenes, such as cineole, (-) selinene, linalool, and geraniol, are abundant in both *Cymbidium* and *Phalaenopsis* orchids [89,90]. The major terpenoids found in an orchid hybrid (*Vanda* Mimi Palmer) are ocimene, linalool, linalool oxide, and nerolidol. 

Since rose is one of the most economically important ornamentals due to its widespread cultivation for cut flowers, essential oil, and perfumes, its floral aroma is the primary economic characteristic, and cultivars are classified based on the concentration of their aromatic components. Monoterpenes are the primary constituent of the floral volatile profile of many rose varieties, including ‘Fragrant Cloud’ and six Hybrid Rugosa roses [91]. 

*Lilium* species and varieties are widely regarded as among the best cut flowers and potted plants in the world, with over a hundred cultivars commercially available based on flower shape and color [92,93]. *Lilium* is highly valued by consumers due to their large showy flower and rich in fragrance, which is primarily composed of terpenoids and benzenoids, with linalool and ocimene being the most prominent among all. Its emission occurs in a circadian rhythm, coinciding with floral visitors [12,13,15,94,95].

*Hedychium* is a fragrant plant that is grown commercially for its ornamental and pharmacological values. *Hedychium* flowers release a broad array of volatile organic compounds, the majority of which are terpenoids (monoterpenes and sesquiterpenes), phenylpropanoids, and fatty acid derivatives [14,96,97,98,99]. *Hedychium* species vary in color and shape, and in terms of floral scent, *Hedychium* species range from scentless to fragrant with high ornamental values [16,27,100,101].

Plants of the lavender genus are known for their exceptional aromatic and therapeutic qualities [102]. Linalool, linalyl acetate, 1,8-cineole, and α-terpineol are the primary volatiles that can be extracted from lavender essential oil [102,103,104]. Other volatile compounds that can be extracted from lavender essential oil include oxygenated derivatives of monoterpenes and monoterpene alcohols [104]. Terpenoids were the main constituents of essential oils in both *Lavandula officinale* and *L*. *angustifolia* [105,106]

*Narcissus* floral volatile compounds and essential oils are extensively being used in the cosmetics industry. The major VOCs in Chinese daffodil flowers (*Narcissus tazetta*) were acetic acid phenethyl ester; ocimene (E-ocimene, allo-ocimene, and neo-allo-ocimene); α-linalool; 1,8 cineole. and benzenoids [107,108,109]. The main VOCs in daffodil (*Narcissus pseudonarcissus*) are monoterpenes, particularly β-myrcene and β-ocimene [110]. Likewise, terpenoids are the primary constituents of floral aroma profiles in Narcissus essential oils [111].

There are only a few fragrant tulip cultivars, but they produce a wide variety of different floral scents. Monoterpenes (linalool, α-pinene, β-ocimene, eucalyptol, and d-limonene), sesquiterpenes (caryophyllene, α-farnesene, β-ionone, and geranyl acetone), and benzenoids are the primary odorants in tulip (*Tulipa* L.) cultivars [112].

Jasmine flowers are famous for their delicate and distinctive aroma profiles. The floral volatile profile of *Jasminum* species (*J*. *grandiflorum*, *J*. *auriculatum*, *J*. *sambac*, and *J*. *multiflorum*) is dominated by monoterpene linalool and sesquiterpene (3*E*, 6*E*)-α-farnesene [113,114]. 

Given the importance of volatile compounds in flavor and health effects on consumers, multiple studies on aroma volatiles of horticultural crops have been conducted. However, there is a need to explore the precise volatile profiles of them through advanced instruments and identify the key genes involved in the specific biosynthetic pathways of these volatiles. Farmers are typically compensated for production quantity rather than flavor or aroma, so less attention is paid to preserving or enhancing desirable fragrance and flavor.

### 2.4. Analytical Protocols and Methods for the Characterization of VOCs

The scientific and industrial communities have recently increased their interest in identifying and quantifying the volatile odorant molecules released by natural products of horticultural commodities. These compounds can be either natural or synthetic, and they are classified based on their sensory properties. In addition to the characteristics and structure of the raw materials, the aroma is also dependent on the chemico–physical properties of the aroma itself, which determines how volatile the odorants are [115]. There are two types of volatile compounds, endogenous and emitted, with the former typically requiring direct organic solvent extraction.

Extraction can be accomplished with a wide variety of solvents, including but not limited to hexane, pentane, diethyl ether, dichloromethane, chloroform, ethyl acetate, and solvent mixtures [116,117]. A solid-phase extraction (SPE) column was used to separate some non-volatile compounds from a volatile organic solvent extract [118]. Headspace sampling techniques such as solid phase microextraction (SPME), Gerstel Twister, and Monotrap, and dynamic headspace sampling systems have been used to collect emitted volatiles [119,120,121,122]. Solid–liquid extractions (microwave-assisted extraction (MAE) and ultrasound-assisted extraction (UAE), accelerated solvent extraction (ASE), supercritical fluid extraction SFE), and solid-phase microextraction are among the new techniques (SPME) [44].Under atmospheric pressure, organic solvents such as hexane, acetone, methanol, ethanol, or water were commonly used, and the solvent of selection was largely determined by the polarity of the analytes. The next step in the process involves identifying the volatiles by using the appropriate analytical tools.

Gas chromatography-mass spectrometry (GC-MS) and headspace analysis have become increasingly popular in recent years for determining the identity and concentration of aromatic compounds in flowering plants. Gas chromatography-mass spectrometry (GC-MS) is a popular and powerful technique for analyzing volatile compounds, and it can provide information on chemical structure [13,100,123,124]. The Electronic nose (E-nose) is another approach that have been widely employed in testing of quality of products, medical diagnosis and environment monitoring [125,126]. The zNose technique, which combines a collection device and an analytical instrument to collect and analyze volatiles in real time, has been established for measuring volatile compounds emitted by plants [119]. Metabolites of the glycolysis and pentose phosphate pathways can be analyzed using mass spectrometry (MS) in conjunction with an isolation technique such as gas chromatography (GC), liquid chromatography (LC), or capillary electrophoresis (CE). The most reliable techniques for determining plant metabolites, such as those involved in the production of volatile fatty acid derivatives, are gas chromatography (GC) and liquid chromatography (LC) or mass spectrometry (MS) coupled with GC or HPLC. GC is the standard method in laboratories across academia and industry for characterizing fatty acid profiles of lipids in biological materials such as human food. Isoprene and linalool have been analyzed by using HPLC-tandem MS (MS/MS) and ultra-HPLC-MS/MS with ion-pair reagents. Fourier transform near-infrared (FT-NIR) spectroscopy with attenuated total reflection (ATR-FT-NIR) or GC-olfactometry (GCO) has been used to assess the sweetness of fresh apples as an internal quality attribute [44]. 

The most common method for identifying fatty acids in horticultural commodities is gas chromatography (GC) coupled to a flame ionization detector (FID) or a mass spectrometer (MS). Although HS-SPME-GC-MS has been widely used for floral aroma detection, combining HS-SPME-GC-MS with Proton transfer reaction mass spectrometry (PTR-MS) or electronic nose (E-nose) would be very efficient for accurate determination of floral volatiles [100,101,127], or it could more proficiently assess the complex volatile phenotype. Through molecular breeding, there are opportunities for aroma enhancement and new applications of the most current innovations in biotechnological modifications of flavor and aroma in horticultural crops.

## 3. Industrial Applications of Plant Volatiles

**Figure 2:**
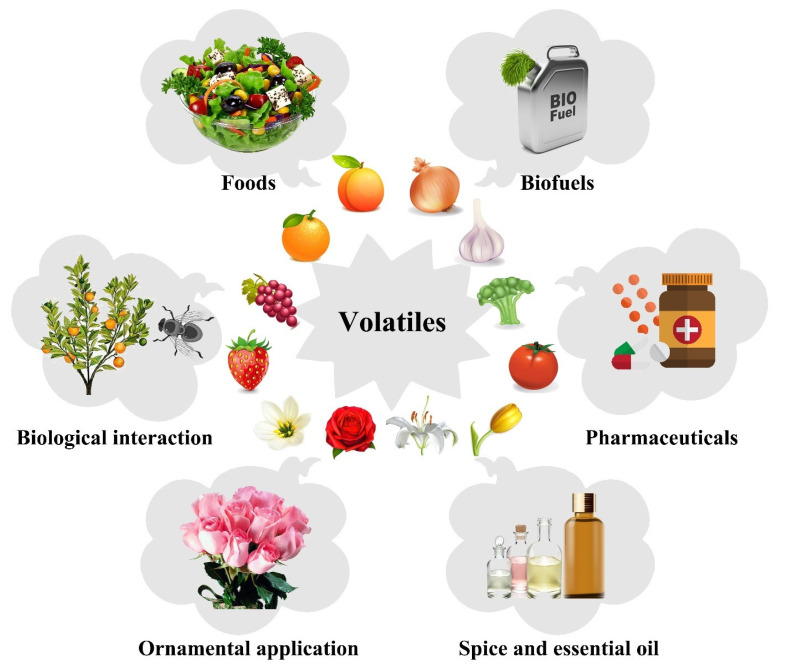
A visual representation of plant volatiles used in plant biological interaction, pharmaceuticals, cosmetics, the food and flavor industry, and biofuels. Biological interactions include both below- and above-ground interactions. Plant volatiles play an important role in multiple plant off-springs and lifespan by attracting pollinators and mediating various interactions between plants and their surroundings.

Aside from their critical role in plant survival, volatile terpenoids have a wide range of important applications in humanity. They are widely used in the pharmaceutical industry for their medicinal properties, in the cosmetic industry for their strong and appealing fragrance, and in the food industry as flavoring agents (Figure 2). The focus of this review is on high-value terpenoids in pharmaceuticals, fragrances and flavors, cosmetics, agriculture, and useful biomaterials.

### 3.1. Food and Flavor Industry

Plants have the ability to generate, accumulate, and release volatiles that, when interacting with human receptors, can act as aroma and flavor molecules. Flavor and aroma are desirable attributes that influence the quality of horticultural crops. To ensure customer perception, a desirable mix of volatile compounds is desired. The olfactory epithelium lining the nasal cavity contains receptors that allow humans to comprehend volatile compounds [128]. Volatile compounds contribute significantly to the distinctive flavors and off-flavors of several foods [129]. Terpenoids are the primary components of most plant essential oils, providing a wide range of pleasant scents, ranging from flowery, fruity, woody or balsamic notes. As a result, terpenoids are a highly valued class of VOCs in the fragrance and flavor industries [130]. 

Flavorants, pharmaceuticals, agricultural pesticides, and chemical industries all rely on volatile compounds for their commercial success. Terpenoids are responsible for a wide range of odors, including “fruity” and “floral”, as well as “earthy” and “woody”. Terpenoids account for more than 90% of the volatiles in citrus peel oil, while in lime, the most copious volatiles were limonene (73.5%), geranial (8.4%), neral (4.9%), myrcene (2.1%), and β-bisabolene (1.6%) [131]. Quantitative analysis of mango fruit found that the most prevalent volatile compounds were monoterpenes such as δ-3-carene, limonene, terpinolene, and β-phellandrene [68]. Over 96% of the orange fruit’s volatiles, which contribute to its flavor, are terpenoids [132]. C13 norisoprenoids, a class of volatiles derived from carotenoids, were found to be the most abundant in raspberry fruit, accounting for between 64% and 94% of the total volatile content across nine different raspberry genotypes [133,134]. Likewise, β-ionone was the major aroma compound in ‘Meeker’ raspberry, with a “raspberry, perfume, floral” aroma [51].

Monoterpenes and sesquiterpenes accounted for 97% of the volatile compounds in seven carrot varieties. These compounds included myrcene, γ-bisabolene, γ-terpinene, α-pinene, sabinene, terpinolene, β-caryophyllene, and limonene [135]. According to Gas chromatography–olfactory detection, foremost terpenes that contribute significantly to fragrance include myrcene, terpinolene, sabinene, and 1,3,8-p-menthatriene [136], having “herbaceous and woody”, “sweet and piney”, “woody and spicy”, and “camphoreous and herbal” aroma notes, respectively [128]. Geosmin, a terpenoid, is responsible for the “earthy” aroma of red beets [137]. Tomatoes get their “fruity” and “floral” aromas from the terpenoids geranial, β-ionone, β-damascenone, and 6 methyl-5-heptene-2-one [138].

Due to their pleasant scents, essential oils (predominantly mono- and sesquiterpenes) derived from aromatic trees, herbs and shrubs such as rose, jasmine, *Hedychium*, *Boswellia* and *Santalum* have attained high global market values. Essential oils of fragrant plant species have been commercially exploited due to their distinctive flavors in the perfume, beverage, and food industries, including valencene (a sesquiterpene) derived from citrus fruits [139]. Limonene, linalool, and 1,8-cineole (monoterpenes) are wildly used for the scent of lime/lemon beverages [21]. Sesquiterpenes (β-caryophyllene and α-humulene) contribute to the aroma and flavor of hops, which influence beer quality [140,141]. 

Genetics, maturation, pre- and postharvest environmental conditions, and postharvest handling all contribute to the distinct chemistry and biochemistry that gives fresh fruits and vegetables their distinct flavor and aroma. Flavor is determined by a dynamic chemical profile that is affected by these factors. Aroma and flavor are imparted to a product by a unique combination of aroma-active volatile compounds, which may include "impact compounds" unique to that product.

As food supplements and colorants, beta-carotene and astaxanthin hold a substantial market value. In strawberries, nerolidol and several key signature terpene synthase genes responsible for the formation of volatiles have been identified and functionally characterized. Likewise, in mango fruit, several terpenes such as caryophyllene, terpinolene, beta-myrcene, 3-carene and alpha-pinene substantially contribute to the mango aroma profile [142]. In carrot, terpene synthases were mainly responsible for the production of aroma and flavor compounds [143]. Quality parameters of foods can thus be enhanced through advanced metabolic/genetic engineering tools. In the coming years, we should have a better grasp on the flavor formation mechanisms especially with the advent of new genomic and metabolomics technologies. Greater consumption and consumer satisfaction can be achieved by developing and implementing methods for enhancing the flavor of fresh horticultural commodities.

### 3.2. Pharmacological Applications

Plants synthesize a variety of volatile organic compounds, and most of them are volatile terpenoids. Terpenoids are being used in the diagnosis and treatment of a variety of human diseases. A large number of terpenoid compounds and derivatives have been evaluated to determine the biochemical and cellular basis of their pharmacological properties [144,145]. 

Floral scents have been shown to have positive effects on mental health, depression, and memory disorders. For instance, plum blossom fragrance, has been shown to have positive effects on mood and may even help train specific regions of the brain responsible for memory, speech, and motor control, which could have positive effects on mental health, depression, and neurological memory disorders [146]. In a natural setting, people are more receptive to the subtle odors of plants, and gardens can be designed to take edge of these appealing floral aromas, which may create feelings that result in various types of therapeutic experiences [147,148]. Floral scents frequently evoke memories of specific times, events, places, or feelings. Aromatherapy is a natural approach of healing the mind, body, and soul. Aromatherapy has been used as an alternative or complementary medicine since ancient times, particularly in ancient Egypt, China, and India [148,149]. Essential oils and fragrance compounds have been used in the treatment of a wide range of mental and physical ailments, including but not limited to: headaches, pain, insomnia, eczema, stress-induced anxiety, depression, and digestive issues, in both conventional and alternative medical systems [150]. Essential oil aromas such as peppermint, jasmine, and ylang-ylang, as well as individual essential oil components (1,8-cineole and menthol), had a significant impact on basic forms of attention behavior [151]. Inhaling enantiomers of limonene and carvone (chiral fragrances) for extended periods of time alters autonomic nervous system characteristics and states of brain activity associated with self-evaluation [152]. According to the findings of these studies, fragrances have both immediate and long-term effects on human mental and physical health.

According to research, both natural monoterpenes and their synthetic derivatives have antiarrhythmic, anti-aggregating, antibacterial, anti-inflammatory, antioxidant, anticancer, anti-spasmodic, antinociceptive, antifungal, antihistaminic, and local anesthetic properties [153]. Terpenoid substances benefit health by interacting with key molecular targets in human and animal physiology by acting as immunostimulants, modulating blood coagulation hemostasis, increasing antioxidant activity, and modulating transcription of genes that control signaling pathways related to different chronic diseases. 

Taxol, a diterpene, was effective against cancer and malaria (artemisinin, a sesquiterpene) [154,155]. Both of these diseases support pharmaceutical industries valued in the billions of dollars that entirely rely on natural products. 

As an antimicrobial agent, cineole, also known as 1,8-cineole or 1,8-cineol (eucalyptol), is a monoterpenoid oxide found in the essential oils of several plants, including eucalyptus, and is widely used to treat respiratory ailments exacerbated by infection [156]. Likewise, eugenol showed anticancer properties as well as quick bactericidal effects on *Salmonella enterica*. Terpineol was found to have high bactericidal activity against *S. aureus* strains, while citronellol, carveol, and geraniol all demonstrated clear bactericidal effects on *Escherichia coli* [157,158]. Several studies have shown that numerous terpene-based VOCs (β-limonene, α-pinene, p-cymene, linalool, β-phellandrene, and terpinenes) present in the essential oils of several trees, shrubs and herbs (*Hedychium*, *Eucalyptus*, *Ocimum*, *Alpinia*, *Citrus*, coniferous trees, rosemary, *Artemisia,* and several others) have antioxidant, sedative-hypnotic and anti-inflammatory properties [21,155]. The aforementioned two examples are pharmaceutical industries valued in the billions of dollars that entirely rely on natural products. 

Terpenes are also essential components of a variety of human nutritional and health care products, i.e., carotenoids and tocopherols, are source of vitamin A, E, and K and coenzyme Q10 [159,160]. *Artemisia annua* is an important ornamental and medicinal plant that is considered to be effective against COVID-19 [161], and terpenoids are the main constituents of this plant. Identification, isolation, and characterization of terpenoid-associated biosynthesis enzymes is the key step for high production of pharmaceutical terpenes. Furthermore, upregulation of genes and transcription factors (TFs) associated in terpenoid biogenesis and suppression of competitive metabolic pathways are effective strategies for enhancing the levels of pharmaceutical terpenoids. 

### 3.3. Cosmetics Industry

Since ancient times, plants and their VOCs have been the primary source of aroma for perfumes and cosmetics. Flowers such as roses, leaves including lavender, fruits, seeds such as anise, some roots (ginger) as well as barks (cinnamon bark), and some woods, such as pines, continue to provide vast quantities of aromatic plant materials for the perfume industry. In 1970, the global market for cosmetics and beauty products was estimated to be $2.4 billion per year, and by 2017, the global beauty and cosmetics industry was valued at more than $500 billion [21,162]. Organic ingredients, specifically terpenes and terpenols, significantly contribute to the overall market value. 

Terpenes and carotenoids are widely used in the cosmetics industry due to their aroma, health benefits, beauty enhancement and antioxidant properties. Terpenes and carotenoids also protect skin from UV radiation and prevent skin wrinkling, aging, and melanogenesis [163]. Alpha-terpineol is used to improve skin permeability and has insecticidal properties [164]. 

Essential oils are a multifaceted mixture of volatile compounds, particularly terpenoids, that have been used since early on in human civilizational history to enhance the physical health and appearance of the human exterior, as well as to protect body parts from environmental damage [165]. Fragrant essential oils are also important perfume additives in cosmetic products. Numerous monoterpenes, including geraniol, ocimene, eucalyptol, citronellol, limonene, camphor, pinenes, linalool, citral, and myrcene, are well-known aromatic compounds found in essential oils of various plants and are widely used in cosmetic and perfumed products [19,166]. Flower essential oils such as gardenia, tuberose, *Lavandula officinalis*, rose, narcissus, and jasmine continue to be among the most prominent aroma components in the cosmetic industry [163]. Linalool is an essential compound in the industrial production of many fragrance substances, including geraniol, nerol, citral, and its derivatives, and a key substance in the synthesis of vitamins A and E [167]. Likewise, limonene is used in common cleaning products. 

There remain scented species whose natural compounds have yet to be elucidated, which could provide a source of new human-beneficial terpenes. Much research remains to be done regarding the several aromatic compounds that have little or no information.

## 4. Metabolic Engineering Strategies for Production of VOCS

Metabolic engineering aims to accurately model biological networks, determine the output of valuable products on an industrial scale in a cost-effective manner, and identify network components that limit the manufacturing capacity of such products [168]. Due to the enormous use and involvement of secondary volatile metabolites in modifying floral aroma profile, enhancing fruit quality, eliminating unwanted compounds, and bettering both direct and indirect plant defenses, metabolic engineering has enormous potential in various agricultural applications [5,169]. For example, pollinators frequently select high floral VOCs in ecological systems, implying that pollination services can be enhanced by generating more fragrant flowers [170,171]. 

**Figure 3:**
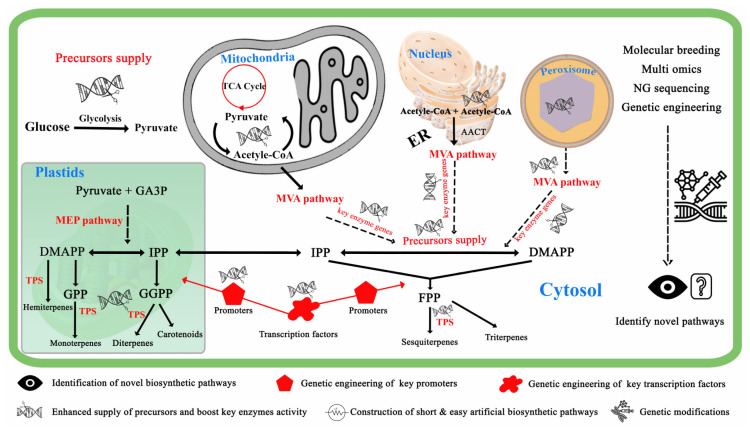
Terpenoids biosynthetic pathways and potential target points for large-scale genetic modification of products. Terpenoids are synthesized via mevalonic acid (MVA) and methylerythritol phosphate (MEP) pathways. The MEP pathway occurs in plastids and begins with pyruvate and GA-3P condensation and proceeds through a series of reactions to yield hemiterpenes, monoterpenes, and sesquiterpenes; the MVA pathway, on the other hand, begins with acetyl-CoA condensation and proceeds through a series of chemical reactions that stretch the cytosol, peroxisomes, and endoplasmic reticulum to yield monoterpenes and sesquiterpenes. Abbreviations: GA3P, Glyceraldehyde 3-phosphate; DMAPP, dimethylallyl diphosphate; IPP, isopentenyl diphosphate; TPS, Terpene synthase; FPP, farnesyl diphosphate; GPP, geranyl diphosphate; GGPP, geranyl geranyl diphosphate; ACoA, acetyl-CoA; ER, endoplasmic reticulum; Per, peroxisome; NG sequencing, Next generation sequencing.

Similarly, flower sizes of many crop cultivars are smaller, and some lack volatiles or have less volatiles, attracting flies that are insufficient pollinators, therefore, molecular breeding and genetic engineering tools may be applied to benefit such crops in attaining those advantageous characteristics. Furthermore, many high-yielding crops, such as corn, lack key defense-related aroma volatiles, such as (*E*)-β-caryophyllene, which attracts pest-killing parasitic wasps and entomopathogenic nematodes [3]. Thus, methodologies to overcoming these issues must be developed, and metabolic engineering of both floral and defense-related VOCs is a valuable technique for enriching plant chemodiversity and beneficial insect biodiversity. Altering biosynthetic pathways is an effective method for improving the flavor, fragrance, and aroma of fruits, herbs, and vegetables (Figure 3). 

### 4.1. Enhanced Terpenoid Bioengineering Approaches

Previously, bioengineering approaches have been applied to boost plant defense mechanisms. In *Arabidopsis*, elevated levels of linalool deterred the aphid (*Myzus persicae*) in dual-choice assays following transcriptional activation of the *F. ananassa* synthase (*FaNES1*) targeted to chloroplasts [5]. Moreover, petunia transformed with the (*S*)-limonene synthase gene from *Clarkia breweri* produced linalool, which repelled aphids [83]. As volatile-based plant defenses are species-specific, findings attained in model systems cannot be readily transferred to crops. Furthermore, pleiotropic impacts of altered VOC profiles on plant defense must be assessed within an agroecosystem setting.

Terpenoid bioengineering efforts may also hold implications for the pharmaceutical properties of plants and affect their ability to treat a variety of ailments. Recently, artemisinin (sesquiterpene) and taxol (diterpene) have been widely used in the pharmaceutical industry [21], and their increased production could benefit humans both economically and physiologically. A major bottleneck in the application of bioengineering technology is the lack of precise and efficient DNA modification tools. However, numerous research findings have shown that upregulation of TPS genes is an efficient strategy for addressing these issues through manipulation of terpenoid production in transgenic plants [4,95,172]. Moreover, as the price of DNA synthesis has decreased, a large number of potential enzymes have been codon optimized, synthesized, and used to complete metabolic pathways. Together with the rapid advancement of plant metabolism, genetics, and plant modern genetic approaches, researchers have advanced from single-celled organisms to sophisticated plant systems. Moreover, plant synthetic biology is a new field that incorporates engineering principles with plant biology [173].

### 4.2. Host Plant/Organism Selection

Countless essential plant natural products pathways have been reconstructed using microbes and model plants. Yeast is typically a more suitable host in synthetic biology approaches for uncovering complex plant volatile metabolic pathways due to its eukaryotic features [174]. Due to their strong biosynthetic ability, microorganisms (*Escherichia coli* and *Saccharomyces cerevisiae*) are broadly used for the biosynthesis of several plant secondary metabolites, and their genetic operation and metabolic modifications have been thoroughly studied [173,175]. Longer biosynthetic pathways, on the other hand, are a limitation in large-scale production of these products, therefore there is a need to identify new pathways and engineer easier, shorter, and more effective pathways to produce a greater quantity of VOCs. The rapid development of high-throughput sequencing has made it feasible to reveal the genetic code of several important species and implement genetic mutation among individuals at the population level, which has served as the foundation for in-depth gene mining and research into plant volatile biosynthesis mechanisms.

### 4.3. Epigenetic Modification

DNA/RNA methylation and histone modification, two epigenetic factors that govern both genes and transcription factors, may stimulate adaptive mechanisms to evolutionary pressures while also regulating the rhythmic emission of VOCs via circadian clock regulation [176]. Recent advancements in omics advancements have facilitated the identification of genes encoding candidate enzymes implicated in the regulation of plant VOCs biosynthesis and a deeper understanding of this process. However, the epigenetics’ function in VOC metabolic pathways and regulation has been disregarded.

Changes in gene function caused by epigenetic regulation are not dependent on a change in DNA sequence but can be passed down through generations in both the mitotic and meiotic cells [177]. Two epigenetic signaling pathways have a significant impact on gene regulation and expression patterns: posttranscriptional modifications involve chromatin transformation, whereas cytosine DNA methylation is implicated with both transpose elements and genes [176]. Methylation alters the biophysical properties of DNA, allowing some proteins to inhibit DNA recognition while enabling others to recognize it, resulting in gene silencing [178]. These methyl-binding proteins are transcriptional suppressors that cause chromatin remodeling by recruiting corepressors and histone deacetylases [179]. Furthermore, chromatin modification and transcriptional silencing can be accomplished using RNA-based epigenetic mechanisms. To a large extent, epigenetics would be responsible for controlling VOC emissions in plants.

Non-coding RNAs have long been recognized for their infrastructure significance [180]. The formation and stability of heterochromatin is thought to be aided by RNA-mediated epigenetic inheritance. In *Schizosaccharomyces pombe*, RNAi machinery disruption causes heterochromatin abnormalities, particularly H3K9 methylation issues [181]. RNAi-mediated DNA methylation is easily recognized in plants, but in humans, this may be confined to the infrequent non-CpG DNA methylation seen largely early stages of development [182]. Non-coding RNAs, both small and long (smRNA and lncRNA), play critical roles in transcription, RNA processing, and protein synthesis [183,184]. In addition to playing a role in the epigenetic mechanisms of histone and DNA methylation patterns, small RNAs also serve as a component of self-reinforcing, positive feedbacks with an amplification element. To date, our understanding of how lncRNA scaffolds independently recruit polycomb-group proteins and other histone modifications complexes is limited.

Given the environmental challenges that plants face and the fact that epigenetic mechanisms are reversible, they may be critical to their survival. Epigenetic variables have lately emerged as important modulators of quick plant responses to surroundings, allowing plants to respond more efficiently to repeating stress events while also preparing progeny for subsequent obstacles [185]. The emission of volatile organic compounds is modified by epigenetic modification of the circadian clock. The expression of genes and transcription factors involved in phenylpropanoid and terpenoid biosynthesis can also be altered by other epigenetic alterations, such as DNA/histone modification and long noncoding RNAs. Furthermore, the wide range of VOCs and their importance in plant evolution can be explained largely by the combination of plant polyploidy and epigenetics.

### 4.4. Overexpression or Silencing of Key Structural Biosynthesis Genes

The preferred strategy for functional verification of target pathways in their native plants has recently been to suppress the function of all or a subset of the enzymes via gene deletion or expression silencing, and afterwards assess changes in terpenoids to detect the functions of candidate pathway genes. Silencing *HcMYB1/2/3/7/8/75/79/145/238/248*, Aux/*IAA* and *HcARF5* significantly reduced the expression level of key volatile terpene synthesis genes, resulting in lower volatile terpenoid emission and indicating that these transcription factors play critical roles in *H. coronarium’s* volatile regulatory mechanism [14,16,27,96,97]. Similarly, Azuma et al., used overexpression/gene silencing and GUS staining assays to identify that *InMYB1* promoter expression is petal-specific and presents a useful tool for molecular breeding of numerous floricultural crops (Eustoma, chrysanthemum, carnation, Japanese gentian, stock, rose, dendrobium and lily) [186]. These findings suggest that transcription factors and promoters could be important targets for modifying crop plant volatile profiles using a combination of genetic engineering tools.

### 4.5. CRISPR/Cas9 Gene-Editing Approach

With the advent of CRISPR/Cas9 in recent years, gene-editing technology has been extensively used for functional validation of key enzymes and regulatory elements due to its versatility and capacity to knock out several genes at once with high accuracy and precision [187,188]. CRISPR/Cas9 genome editing has been applied to many plants for gene expression regulation and enzyme manipulation. Strong fragrance, for example, was successfully developed in non-aromatic rice by creating novel alleles of *OsBADH2* through genome editing with CRISPR/Cas9 [189,190]. Moreover, using synthetic biology platforms to elucidate such pathways has also proven an effective method.

### 4.6. Subcellular Compartmentation

Targeting subcellular compartments may be an effective strategy for increasing plant volatile production, such as the introduction of artemisinin in tobacco (*N. tabacum*) into the chloroplast and nuclear genomes using two distinct biosynthetic pathways, which increased artemisinin production without interfering with plant physiology. Researchers manufactured high level terpenoid production in tobacco plants by upregulating the expression of IPP (isopentenyl diphosphate) and diverting carbon flow from the cytosolic or plastidic compartments, resulting in 1000-fold increases in production of amorpha-4,11-diene and patchoulol (sesquiterpenes), and 10–30-fold increases in limonene (monoterpene) production [191,192,193,194].

Chemical synthesis is frequently inefficient and costly, and it may not produce enantiomerically pure terpenes, whilst large-scale microbial manufacturing involves the use of expensive feedstocks. The large-scale production of VOCs naturally in plants via metabolic engineering approaches may provide an optimal avenue to meet the demands of both therapeutic applications and industrial production.

## 5. Conclusions

The volatile compounds produced by plants have profound effects on all forms of life. Flowers and fruits release aromas to attract pollinators and humans who consume the fruit. Most horticultural varieties and cultivars, on the other hand, are chosen based on human preference. It is critical to identify VOCs that are relevant to human sensory preferences in order to meet consumer requirements. Flower and fruit aromas have been extensively studied from the perspective of plant ecology, but their application to humans has received less attention. Recent technological advances have accelerated the discovery of VOCs and the synthetic enzymes, genes, and TFs responsible for their synthesis, resulting in a better overall understanding of VOC biosynthesis in plants. There are, however, a number of unanswered questions that must be addressed. Its role can be broadened with the help of genetic engineering by improving crop plant genetic ability, improving the aroma quality of fruits and flowers, and producing pharmaceuticals.

## 6. Research Gaps and Future Directions

Plants can produce a wide range of volatile metabolites that attract pollinators and seed dispersers while also strengthening plant defense responses. Maximizing plant volatile production is also beneficial to the cosmetics, pharmaceutical, food and flavor industries. Furthermore, metabolic engineering can also be used to develop crops with enhanced defense systems, lowering the agricultural sector’s reliance on harmful pesticides. The use of data mining algorithms to process information on VOC emissions as well as environmental variables obtained from fields by various sensors will enable the utilization of big data to evaluate plant performance and determine early signs of stress. The goal of metabolic engineering is to create precise models of biological networks in order to predict the yield of economically valuable products at industrial scale and to pinpoint the elements of these networks that restrict production.

Although several volatile biosynthesis pathway genes have recently been identified, the mechanism underlying aroma and flavor of several crops remains largely unknown. Decoding genome sequencing of numerous plant species has recently been accomplished, providing an opportunity to identify and characterize key signature genes associated with aroma production, thus improving the potential of genetic engineering to successfully enhance aroma quality of horticultural commodities. Through such endeavors, plant genetic engineering has the potential to yield aromatic horticultural products that are high in health-promoting phytonutrients.

Although the biosynthetic pathways of volatile formation have been studied, there is still a need to reengineer the existing pathways using metabolic and genetic engineering, as well as to investigate potential novel unknown pathways and biosynthesis genes, which has recently become possible with the advancement of innovative omics and genomic tools. Correspondingly, there is an urgent need to determine which transcription factors control the production of these fugitive compounds. Unfortunately, the role of various hormones (such as auxin, abscisic acid, salicylic acid, ethylene, and jasmonic acid) and environmental factors (such as temperature, light, humidity, and so on) in influencing aroma quality is understudied. There is also a lack of information about the TFs that are involved in hormonal or environmental signaling pathways. A better understanding of how hormones, environmental factors, and their interplay regulate transcription is a priority for future studies. Gene editing and the discovery of model organisms for enhanced volatile production hold the promise of future benefits for humankind. CRISPR/Cas9 genome editing has made it easy to silence or overexpress specific genes in charge of modifying desirable traits, and it will persist to play a crucial role across every stage of crop plant development, from the introduction of novel biosynthetic pathways to the alteration of floral fragrance in scentless crops. Despite their potentially enormous impact on plant growth, miRNAs and posttranscriptional mechanisms in plants have received comparatively little attention. Understanding the mechanisms underlying transcriptional regulation of active pharmaceutical ingredient biosynthesis via epigenomics, phenomics, and cutting-edge bioinformatics provides a theoretical groundwork for breeding new plant varieties with elevated levels of desirable natural compounds.

Improved volatile production is one example of a novel quality trait that can be incorporated into future germplasm development due to the increased resources available to breeders today. This is aided by the fast development of plant genome sequences, as well as a multitude of aroma- and flavor-associated microsatellites that can be used to identify the most interesting alleles or key signature candidate genes. Investigation of natural diversity and identification of signature volatiles associated with specific plant aroma can provide useful insight for achieving the objectives of enhancing the aroma profile of certain horticultural crops, providing a tangible benefit for end-consumers.

## Data Availability

Not applicable.
